# Co-expression analysis reveals dysregulated miRNAs and miRNA-mRNA interactions in the development of contrast-induced acute kidney injury

**DOI:** 10.1371/journal.pone.0218574

**Published:** 2019-07-15

**Authors:** Zhiqing Wang, Weiwei Bao, Xiaobiao Zou, Ping Tan, Hao Chen, Cancan Lai, Donglin Liu, Zhurong Luo, Mingfang Huang

**Affiliations:** 1 Department of Cardiology, 900 Hospital of the Joint Logistics Team, Fujian Medical University, Fuzhou, China; 2 Faculty of Graduate Studies, Bengbu Medical College, Bengbu, China; 3 Department of Cadre Health Care, 900 Hospital of the Joint Logistics Team, Fujian Medical University, Fuzhou, China; Medizinische Universitat Graz, AUSTRIA

## Abstract

The pathogenesis of contrast-induced acute kidney injury (CI-AKI) is incompletely understood. MicroRNAs (miRNAs) are important mediators that normally function via post-transcriptional degradation of target mRNAs. Emerging evidence indicates the appearance of differentially expressed (DE) miRNAs in CI-AKI following the injection of intravenous contrast medium. However, there are differences in the pathological mechanism and incidence of CI-AKI between intravenous and intra-arterial contrast administration. The present study aimed to investigate the critical roles of dysregulated miRNAs and their associated mRNAs in kidney injury following intra-arterial contrast medium exposure. Based on a reliable CI-AKI rat model, we conducted genome-wide miRNA and mRNA expression profiling analysis using deep sequencing. In the study, 36 DE mature miRNAs were identified (fold change > 1.5 and p value < 0.05) in the kidneys of CI-AKI rats (n = 3) compared with that in the controls (n = 3), consisting of 23 up-regulated and 13 down-regulated DE miRNAs. Bioinformatic analysis revealed that wingnut (Wnt), transforming growth factor beta (TGF-β), and 5'-AMP-activated protein kinase (AMPK) signaling pathways were most likely to be modulated by these dysregulated miRNAs. Around 453 dysregulated genes (fold change > 2.0 and p value < 0.05) were identified. Integrated analysis revealed 2037 putative miRNA-mRNA pairs with negative correlations. Among them, 6 DE miRNAs and 13 genes were selected for further quantitative real-time reverse transcription polymerase chain reaction validation (n = 6 for each group), and a good correspondence between the two techniques was observed. In conclusion, the present study provided evidence of miRNA-mRNA interactions in the development of kidney injury following an intra-arterial contrast injection. These findings provide insights into the underlying mechanisms of CI-AKI.

## Introduction

Contrast-induced acute kidney injury (CI-AKI) is an iatrogenic complication that frequently develops after cardiac catheterization procedures involving the administration of iodinated contrast media (CM). CI-AKI has emerged as one of the most important causes of hospital-acquired acute renal failure, accounting for 10–25% of all cases [1–3]. The onset of CI-AKI is significantly associated with adverse events, including increased mortality and a long-term decline in kidney function [4–6]. Despite advances in the introduction of safer CM and the optimized of hydration protocols, CM continues to pose a substantial threat of CI-AKI in vulnerable renal patients [7]. Research has identified several potential routes for the pathogenesis of CI-AKI, including direct cytotoxic effects, medullary hypoxia, and the perturbation of renal tubulodynamics [8]. However, the precise molecular pathways remain poorly understood, resulting in the absence of specific preventive strategies or sensitive biomarkers for early diagnosis.

MicroRNAs (miRNAs) are a class of evolutionarily conserved endogenous small non-coding RNAs (~22 nt), which normally regulate the stability and translational efficiency of target messenger RNAs (mRNAs) by binding to the complementary sequence [9, 10]. It is estimated that over 60% of mRNAs are regulated by miRNAs, and one single miRNA may modulate more than one hundred mRNAs [11]. A growing body of evidence has demonstrated that these tiny molecules control diverse cellular processes, from development to diseases [10, 12–15] by interfering with gene expression and downstream signaling cascades [16].

MiRNAs are reported to have critical roles in the development of kidney diseases, including acute kidney injury [17–21]. For instance, miR-494 and miR-21 are involved in apoptotic and inflammatory pathways in ischemia-reperfusion models of kidney injury [18–20]. In addition, miR-34a shows protective effects on cell survival during cisplatin nephrotoxicity [21]. Moreover, studies concerning miRNA expression in CI-AKI have been carried out and the levels of several miRNAs, including miR-188-5p, miR-30a, miR-30c, and miR-30e, have been identified as elevated in the plasma and kidneys of rat models [22, 23]. However, we noted that CM was administered via the tail vein in these models, which is different from the clinical practice of coronary angiography. As reported in previous studies, the incidence of CI-AKI is substantially higher following intra-arterial CM injection compared with that induced by intravenous CM injection [24, 25], largely because of the dilution effect of the blood on the CM before the kidney is exposed. To the best of our knowledge, the expression profiles of miRNAs in CI-AKI after intra-arterial CM administration have not been explored.

In the present study, a reliable rat model of CI-AKI that is mostly analogous the clinical condition of arteriography was developed with a significant rise in serum creatinine (SCr) over baseline, which was based on a previously described method with some modifications [26]. The differentially expressed (DE) miRNAs, as well as mRNAs, in the renal tissue between CI-KAI rats and controls were identified using state-of-the-art RNA-sequencing technology. The key miRNA-mRNA communication networks were then illustrated using integrated miRNA-mRNA functional analysis. The candidate miRNAs and mRNAs were then rigorously validated using quantitative real-time reverse transcription polymerase chain reaction validation (RT-qPCR). This study was undertaken to address miRNA-mRNA modulation of the development of CI-AKI.

## Materials and methods

### Materials and animals

Indomethacin and N-ω nitro-L-arginine methyl ester (L-NAME) were purchased from Sigma Chemical Co. (St. Louis, MO, USA). Indomethacin, an inhibitor of prostaglandin synthesis, was dissolved in phosphate buffer (pH 8.4) at 5 mg/ml. L-NAME, an inhibitor of nitric oxide synthesis, was dissolved in 0.9% normal saline at 10 mg/ml immediately before injection. The combined inhibition of the synthesis of prostaglandin by indomethacin and nitric oxide by L-NAME may induce renal vasoconstriction and predispose the kidney to injury. Iopromide (Ultravist 370; 370 mg/ml iodine), a nonionic monomeric low-osmolarity CM widely used in clinical practice, was obtained from Bayer Co. (Leverkusen, Germany). We used 3-month-old male Sprague-Dawley rats (Fujian Medical University breed, China), weighing approximately 300–400 g at the start of the experiment, which were kept in individual cages under controlled conditions of light (12 hours/12 hours light/dark cycle) and temperature (21–23°C), with free access to tap water and standard rat chow for least 7-days for adaption. The animal protocols were conducted according to the Guiding Principles in the Use of Animals of 900 Hospital of the Joint Logistics Team, and were approved by the animal experiment ethics review committees of 900 Hospital of the Joint Logistics Team (Approval number IACUC-2017-17).

### Rat model of CI-AKI and sample collection

Rats were deprived of water for 48 hours and then deeply anaesthetized using pentobarbital sodium (40 mg/kg, i.p.). The right femoral vein and carotid artery were cannulated using short peripheral catheters that are widely used in nursing practice (24 G × 21 mm, SPECATH, Foshan, China), and a baseline blood sample was drawn (1 ml) from the carotid artery to determine the SCr level. Then, indomethacin (5 mg/ml) was administrated intravenously at a dose of 10 mg/kg. Fifteen minutes later, L-NAME (10 mg/ml) at a dose of 10 mg/kg was administered. After another 15 minutes, the rats were randomized to receive iopromide (CI-AKI group, n = 9) or normal saline (Control group, n = 9) at a dose of 7.8 ml/kg via the carotid artery cannulation over a time course of 5 minutes. The rats were then allowed to recover in individual cages with free access to tap water and standard chow. At 12 hours post-operation, a second set of arterial blood samples was obtained under anesthesia. Thereafter, both kidneys were harvested and bisected longitudinally immediately after removing blood cells by perfusing with 0.9% normal saline through the bilateral renal arteries. Then, the rats were euthanized with an overdose of pentobarbital anesthesia (200 mg/kg, i.p.). The levels of SCr were measured at the hospital clinical laboratory using a standard method. One-half of the left kidney, containing the cortex and medulla, was sliced and fixed in the RNAsafety Reagent (Bohao, Shanghai, China) for RNA isolation. The RNA samples were then transferred to a −20°C freezer until use. The right kidney, containing the cortex and medulla, was fixed in 10% neutral formalin liquid for histological analysis. The histological samples were embedded in paraffin, sectioned at 3 to 4 μm thick, visualized using hematoxylin and eosin (H&E) staining, and then evaluated under a light microscope. The slides of each sample were reviewed blindly, and the Paller score was calculated to determine the severity of tubular injury [27]. Detailed laboratory protocols have been deposited in protocols.io (http://dx.doi.org/10.17504/protocols.io.z6bf9an).

Continuous data, such as SCr and the Paller score, are presented as mean ± standard deviation (SD). Comparisons of means between the two groups were performed using a two-tailed Student's t-test. A *P*-value < 0.05 was considered significant.

### Extraction and quantification of total RNA

Total RNA was extracted from kidney tissue samples using a mirVana miRNA Isolation Kit (Ambion, Austin TX, USA) following the manufacturers' instructions. The quantity and integrity of the total RNA were measured using a Nanodrop spectrophotometer (ND-1000, Nanodrop Technologies, Thermo Fisher Scientific, Waltham, MA, USA) and an Agilent 2100 Bioanalyzer (Agilent, Santa Clara, CA, USA). The concentration of total RNA ranged from 942 to 2170 ng/μl. Samples with an RNA Integrity Number ≥ 7.0 and a 28s:18s ratio ≥ 0.7 were eligible for sequencing analysis of miRNAs and mRNAs, and for validation by RT-qPCR.

### Small RNA sequencing and functional analysis

Small RNA libraries were prepared using a Illumina TruSeq Small RNA Sample Preparation Kit (Illumina Inc., San Diego, CA, USA). The 18–44 nt RNAs from the total RNA samples were purified using 15% polyacrylamide gel electrophoresis. These small RNAs were ligated with the 5’ and 3’ RNA adaptors, and then purified using polyacrylamide gel electrophoresis. After first-strand reverse transcription and high-fidelity PCR amplification, a Qubit 2.0 Fluorometer and Agilent 2100 system were used to determine the quality and size of the library, respectively. The final libraries were then submitted for miRNA sequencing on the Illumina HiSeq 2500 platform (Illumina Inc., San Diego, CA, USA). To identify the miRNAs, the clean reads were mapped to the reference genome (ftp://ftp.ensembl.org/pub/release-83/fasta/rattus_norvegicus/dna/Rattus_norvegicus.Rnor_6.0.dna_rm.toplevel.fa.gz) using Bowtie [28], with only one base mismatch allowed. After excluding reads matching tRNAs, rRNAs, snRNA, and snoRNAs, reads matching annotated miRNAs were retrieved from miRbase (version 21.0; http://mirbase.org/). The unmatched reads were analyzed using miRCat (http://srna-workbench.cmp.uea.ac.uk/tools/analysis-tools-/mircat) to predict novel miRNAs. The Bioconductor package, edgeR, was used for differential expression analysis in the miRNA sequencing data [29]. The DE miRNAs between the CI-AKI rats and the controls were determined according to a fold change ≥ 1.5 and a *P*-value < 0.05.

Putative target genes of the candidate miRNAs were predicted using the miRanda database [30]. The miRanda algorithm is mainly based on these three aspects: Sequence complementarity, binding energy of the miRNA-target duplex, and the evolutionary conservation of the target site. Alignment score and free energy were selected as the sorting criteria for conserved target sites, with thresholds of S > 90 and △G < 17 kcal/mol. On the condition that multiple miRNAs targeted the same site on a transcript, the Greedy algorithm was applied, and only the miRNA with the highest alignment score and the lowest energy was reported [31]. The target genes of known miRNAs were then annotated using miRTarBase [32]. To better understand the DE miRNAs and their putative targets, gene ontology (GO) term analysis was performed for functional classification, including biological process, cellular component and molecular function [33]. Association of the putative targets with different pathways was analyzed using the Kyoto Encyclopedia of Genes and Genomes (KEGG) database [34]. The hypergeometric test was performed to identify of the significant GO terms and KEGG pathways using the criteria of a *P*-value < 0.05.

### mRNA sequencing and data analysis

To construct mRNA libraries, total RNA of each sample was assessed for fragmentation. RNA-sequencing (RNA-seq) libraries were prepared after ribosomal depletion using the VAHTS Total RNA-Seq (H/M/R) Library Preparation Kit, according to the manufacturer’s recommendations (Vazyme, Nanjing, China). Following cDNA preparation, the samples were sequenced using the paired end read method (2 × 100 bp) on the Illumina HiSeq X Ten platform (Illumina Inc., San Diego, CA, USA). For mRNA expression calculation, the fragments per kilobase of transcript per million mapped reads (FPKM) method was used in the StringTie and trimmed mean of M values algorithms [35]. EdgeR was used for differential expression analysis in the mRNA sequencing data [29]. The DE mRNAs were determined using a greater than 2.0-fold change and a *P*-value < 0.05.

### miRNA-mRNA integrated analysis

Integrated functional network analysis of the normalized miRNA and mRNA sequencing data was performed according to the RNAhybrid (http://bibiserv.techfak.uni-bielefeld.de/rnahybrid/) and Targetscan databases (http://www.targetscan.org/). Generally, the miRNA-mRNA pairs were expected to be negatively correlated, because miRNAs function to down-regulate their target mRNAs. Pearson correlation coefficients were calculated between each miRNA and its target mRNA. The mRNAs that were significantly anticorrelated with particular miRNAs were selected, with a *P*-value of the correlation coefficient < 0.05. The miRNA-mRNA integrated networks were then presented using Cytoscape [36].

### Validation using RT-qPCR

The candidate DE miRNAs and mRNAs were quantified using Taqman miRNA assays (Applied Biosystems, Carlsbad, CA, USA) following the manufacturer’s protocol. DNA-free RNA samples were extracted as mentioned above. All the specific primers were designed and synthesized using Primer 5.0. RT-qPCR was carried out in triplicate using a miScript II RT kit (QIAGEN, Hilden, Germany) and a 7500 Real-Time PCR System (ABI, Foster City, CA, USA). The U6 small nuclear RNA and the *Gapdh* gene (encoding glyceraldehyde-3-phosphate dehydrogenase) were used as internal control for miRNAs and mRNAs, respectively. The expression levels of miRNAs and mRNAs were normalized and quantified using the 2^-ΔΔ Ct^ method [37]. The relative levels of miRNAs and mRNAs were presented as the mean ± SD. Statistical analysis was performed using Student’s t test, and a *P*-value < 0.05 was considered significant.

## Results

### Functional and histopathological observation

**Fig 1:**
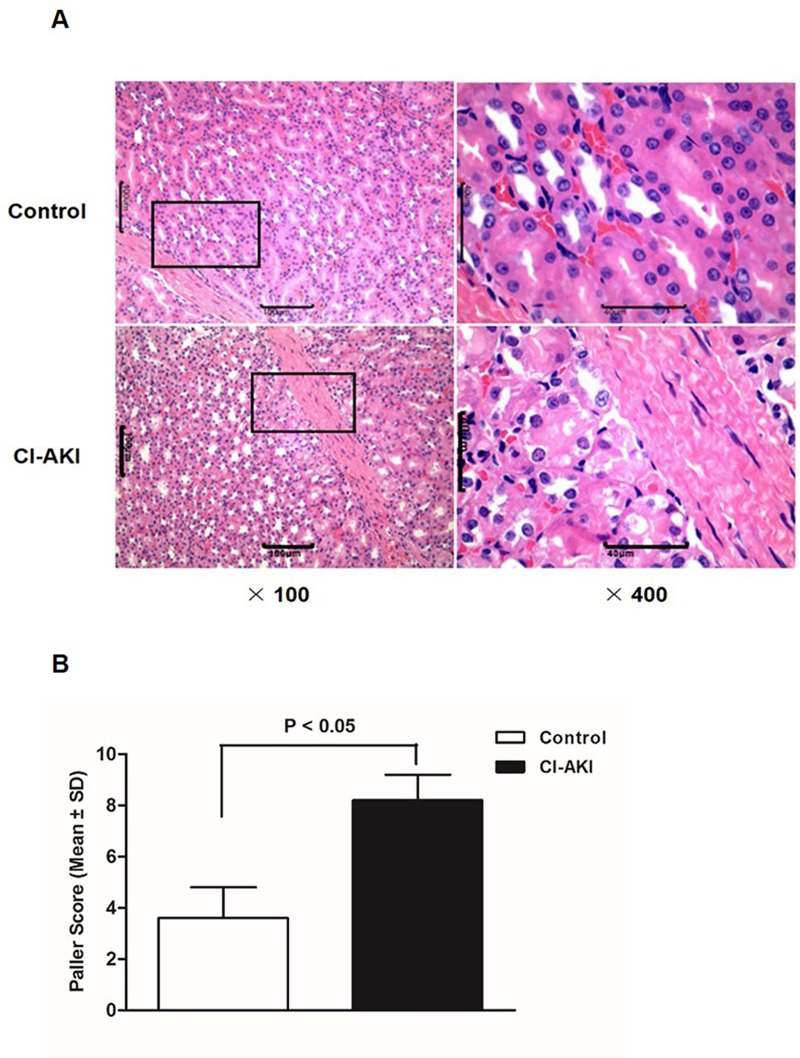
Histopathological assessment of the kidneys of the control rats and CI-AKI rats by H&E staining. (A) CM treatment induces representative histopathological changes in the outer medulla of H&E-stained kidney sections. (B) Tubular injury is graded by the Paller score, a semi-quantitative evaluation. For each kidney section, 100 tubules from 10 highmagnification (×200) fields of the inner and outer medulla are scored. The average Paller score is much higher in CI-AKI rats than controls ((8.2 ± 0.9) vs. (3.6 ± 1.2), *P* < 0.05).

A total of eighteen male Sprague-Dawley rats were randomly divided into the CI-AKI group (n = 9) and control group (n = 9). No significant difference was observed for body weight or SCr levels before contrast injection between the groups (Table 1 and S1 Table, *P* > 0.05). As expected, the administration of CM, following pretreatment by indomethacin and L-NAME, induced remarkable renal dysfunction at 12 hours post-procedure. The average elevation of SCr was (59.9 ± 23.0) % over baseline in the CI-AKI rats, compared with (-10.5 ± 17.1) % in the control rats injected with normal saline (Table 1, *P* < 0.05). Pronounced histopathological alterations in the tubular epithelial cells of the inner and outer medulla were observed in the CI-AKI rats, including vacuole-like denaturation, nucleus pyknosis, and apoptotic body formation (Fig 1A). These pathological findings were consistent with those of previous studies [38, 39]. Additionally, the mean Paller score for the CM- treated kidneys was 8.2 ± 0.9, whereas that for the controls was 3.6 ± 1.2 (Fig 1B, *P* < 0.05), indicating a greater extent of tubular injury after CM intervention.

**Table 1:** Baseline characteristics of experimental groups and the effects of contrast medium on changes in SCr elevation.

Group	Body weight (g)	SCr (μmol/L)		Percent increment of SCr (%)
		Baseline	End	
Control (n = 9)	370.8 ± 28.3	53.3 ± 9.2	47.9 ± 13.1	-10.5 ± 17.1
CI-AKI (n = 9)	342.2 ± 36.1	43.9 ± 12.3	69.5 ± 18.7*	59.9 ± 23.0*

Data are mean ± SD. Statistical analysis was performed using Student’s t test.

*P < 0.05 vs. control group.

### Identification of DE miRNAs

**Fig 2:**
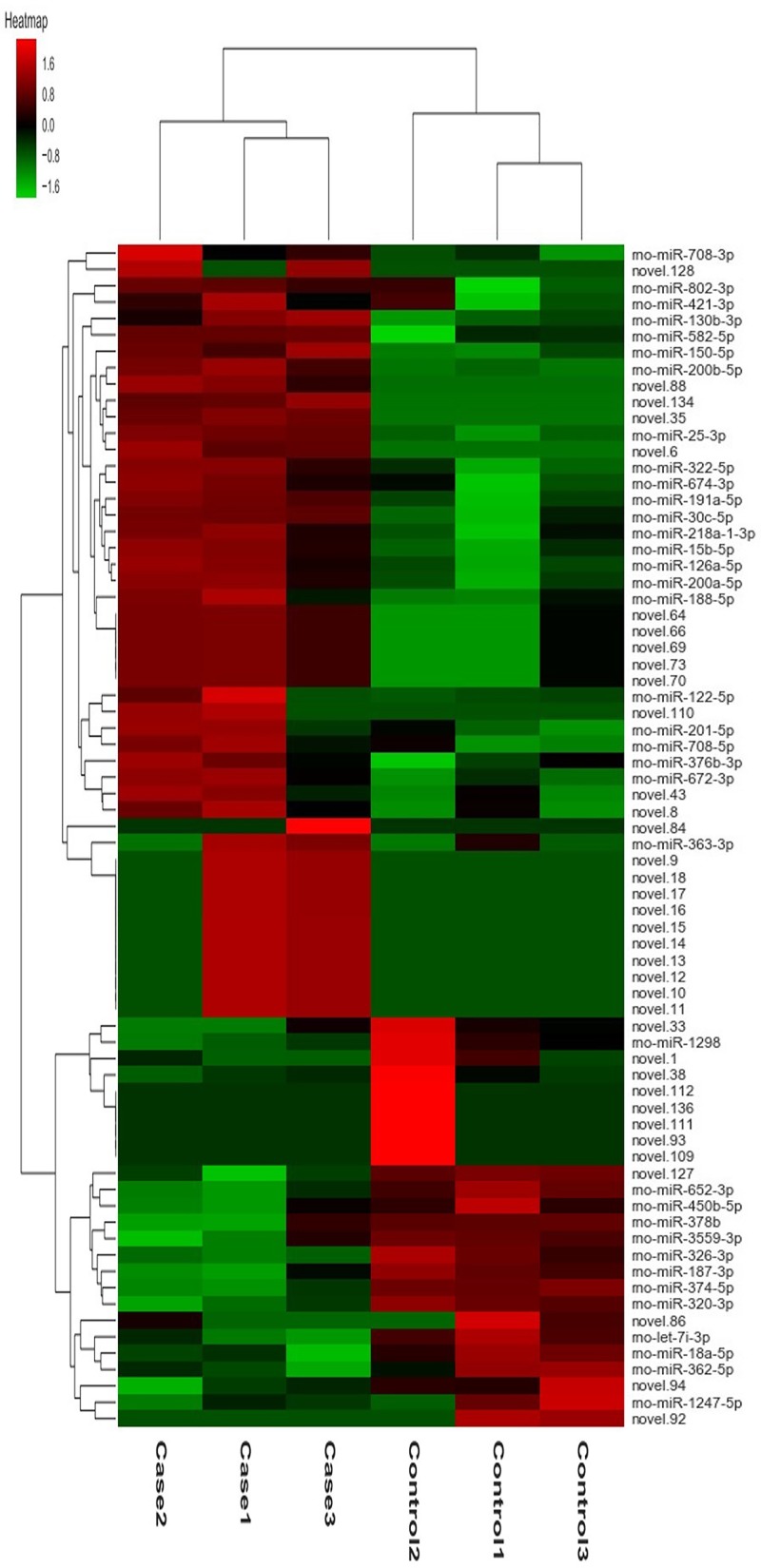
Hierarchical clustering analysis of the 72 DE miRNAs in the CI-AKI and control group. Each group includes 3 duplicates. The DE miRNAs includes 36 mature and 36 novel ones (*P* < 0.05). Colors from green to red represent the miRNA expression abundance from poor to rich.

In the miRNA expression profiling by deep sequencing, six rats were selected from the CM group (n = 3) and the control group (n = 3), respectively. The raw data were deposited in the NCBI Gene Expression Omnibus database (https://www.ncbi.nlm.nih.gov/geo/query/acc.cgi?acc=GSE130796). We identified that 72 out of 673 miRNAs were aberrantly expressed in the CI-AKI rat model compared with that in the control group, including 36 mature miRNAs and 36 novel miRNAs (S2 Table). Hierarchical clustering analysis of the DE miRNAs with the highest variability is shown as a heat-map in Fig 2. As shown in Table 2, we subsequently focused on the 36 mature miRNAs, among which 23 were upregulated and 13 were downregulated. Among them, rno-miR-30c-5p, rno-miR-191a-5p, and rno-miR-126a-5p were the most abundant in the kidneys of CI-AKI rats. Rno-miR-122-5p was overexpressed with the highest fold change of 42.9, while rno-miR-1298 was downregulated with the highest fold change of 9.6. Notably, rno-miR-188-5p and rno-miR-30c-5p had been identified as dysregulated in rodent kidney tissues following intravenous CM injection [22, 23]. Three miRNAs (rno-miR-126a-5p, rno-miR-363-3p, and rno-miR-674-3p) were found previously to be upregulated in the plasma of CI-AKI rats [22]. However, the majority of these mature miRNAs had not been linked previously to CI-AKI according in the published literature.

**Table 2:** List of DE mature miRNAs in kidneys of CI-AKI rats.

Upregulated miRNAs	Fold change	*P* value	Downregulated miRNAs	Fold change	*P* value
rno-miR-122-5p	42.92	<0.001	rno-miR-1298	-9.61	0.042
rno-miR-126a-5p	2.60	<0.001	rno-miR-378b	-2.39	0.004
rno-miR-376b-3p	2.45	0.011	rno-miR-362-5p	-2.26	0.037
rno-miR-322-5p	2.41	<0.001	rno-miR-374-5p	-2.17	<0.001
rno-miR-200a-5p	2.24	<0.001	rno-miR-1247-5p	-2.00	0.007
rno-miR-708-5p	2.08	0.005	rno-miR-18a-5p	-1.71	0.005
rno-miR-363-3p	1.94	0.020	rno-let-7i-3p	-1.68	0.002
rno-miR-708-3p	1.93	0.015	rno-miR-326-3p	-1.68	<0.001
rno-miR-201-5p	1.90	0.002	rno-miR-652-3p	-1.68	<0.001
rno-miR-672-3p	1.89	0.038	rno-miR-187-3p	-1.66	0.002
rno-miR-802-3p	1.87	0.013	rno-miR-450b-5p	-1.62	0.003
rno-miR-191a-5p	1.82	<0.001	rno-miR-320-3p	-1.53	<0.001
rno-miR-200b-5p	1.75	<0.001	rno-miR-3559-3p	-1.51	0.031
rno-miR-25-3p	1.73	<0.001			
rno-miR-218a-1-3p	1.72	0.007			
rno-miR-188-5p	1.67	0.001			
rno-miR-30c-5p	1.67	<0.001			
rno-miR-15b-5p	1.63	<0.001			
rno-miR-421-3p	1.59	0.035			
rno-miR-150-5p	1.57	<0.001			
rno-miR-674-3p	1.57	0.009			
rno-miR-130b-3p	1.53	0.006			
rno-miR-582-5p	1.52	0.017			

**Fig 3:**
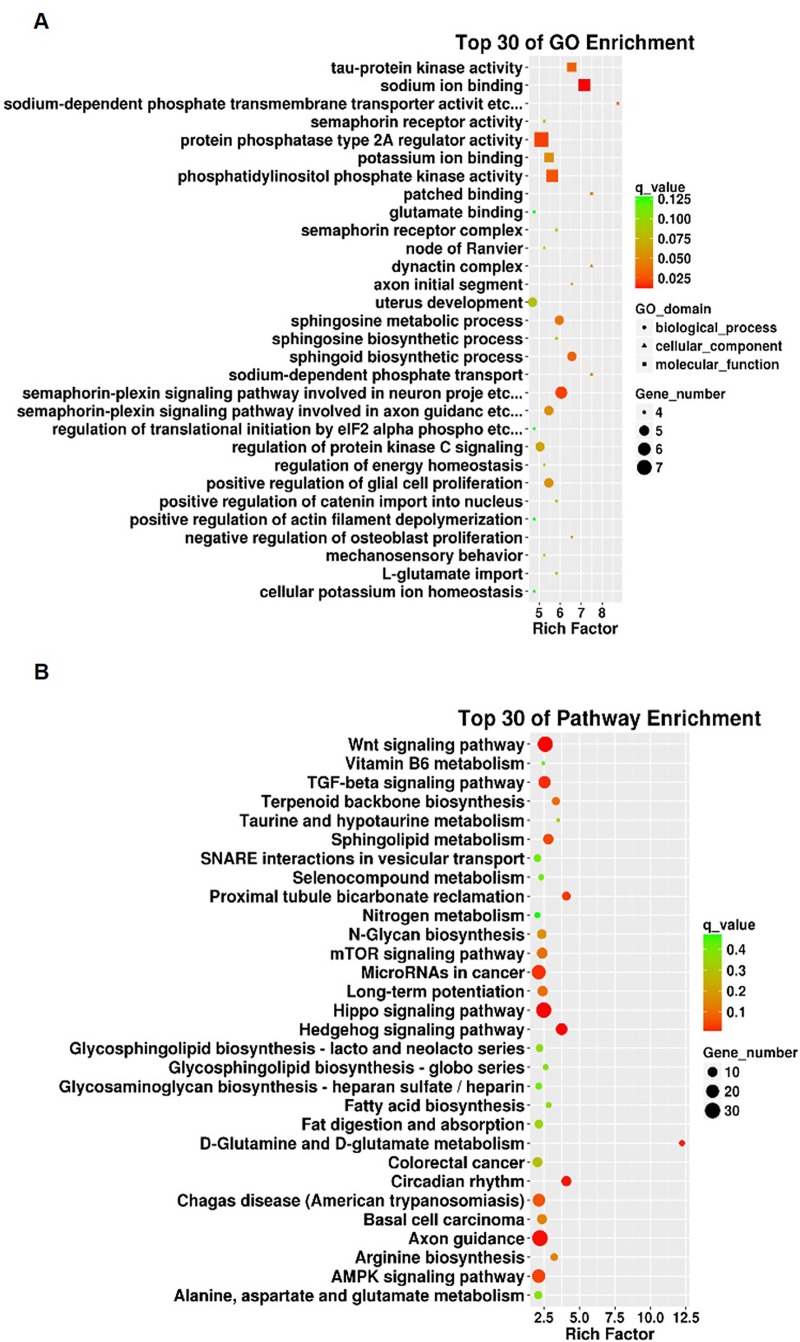
Functional enrichment analysis of the mature DE miRNAs and their putative targets. (A) The enriched top 30 GO terms (*P* < 0.05). (B) The enriched top 30 pathways of KEGG analysis (*P* < 0.05).

To understand the biological and functional implications of these miRNAs and their putative targets in the development of CI-AKI in depth, GO functional enrichment and KEGG pathway analyses were conducted. In the GO analysis results, the enriched categories included 818 different “biological processes”, 106 different “cellular components”, and 148 different “molecular functions” (S3 Table). Thirty most significantly enriched GO terms are shown in Fig 3A. The transcripts were further assembled in 36 potential pathways in the KEGG analyses (S4 Table), and the top 30 are shown in Fig 3B. Generally, “signal transduction”, “immune system”, and “cell growth and death” were identified as three noteworthy categories. Among the canonical pathways were the “Wnt signaling pathway”, “TGF-β signaling pathway”, and “AMPK signaling pathway”. These pathways are liable to be involved in inflammatory and adaptive responses following kidney injury [40–42].

### Identification of DE mRNAs

**Fig 4:**
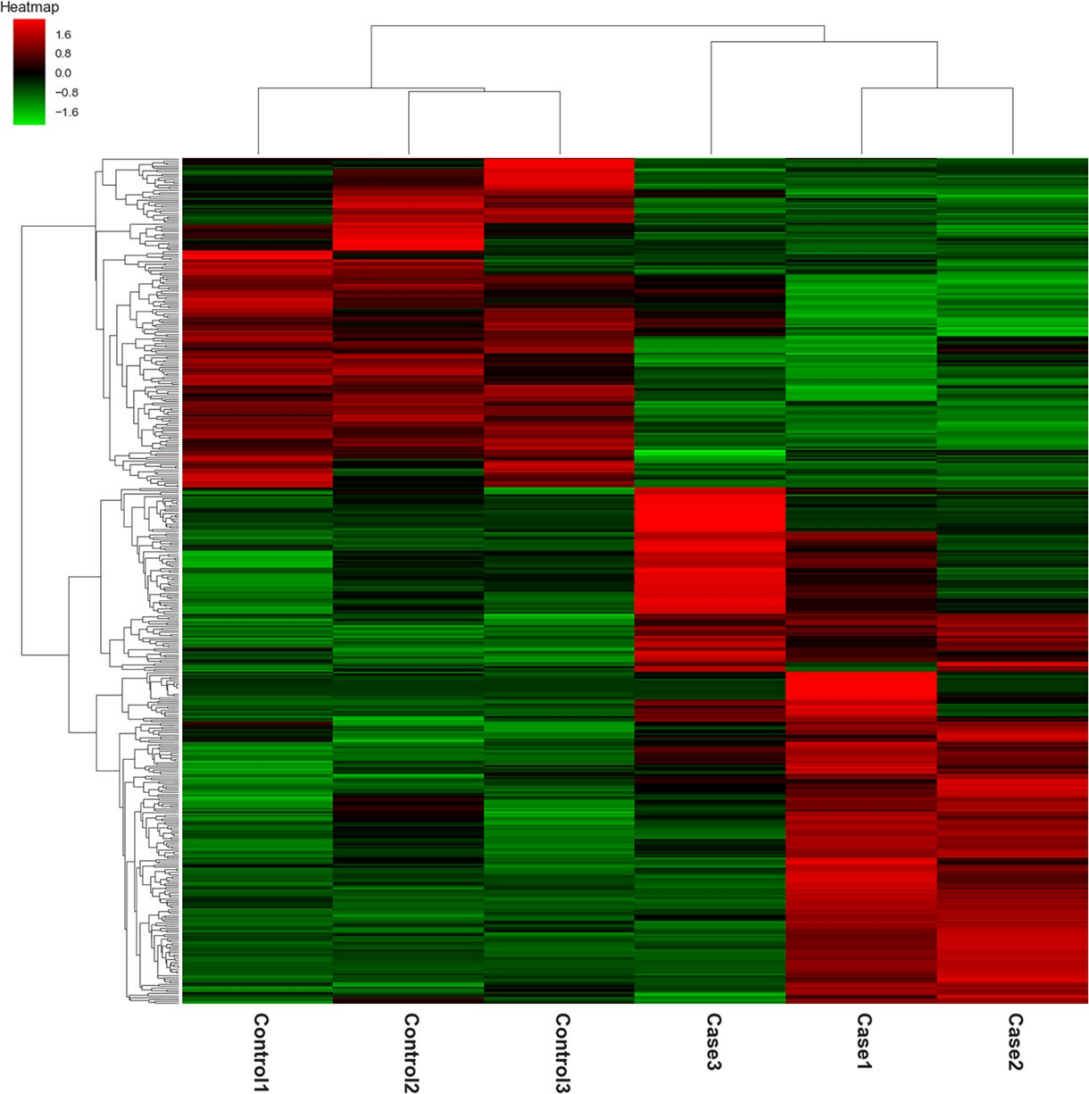
Hierarchical clustering analysis of the 453 DE mRNAs in the CI-AKI and control group (*P* < 0.05). Each group includes 3 duplicates. Colors from green to red represent the gene expression abundance from poor to rich.

Global mRNA expression profiling was performed on three CI-AKI specimens and three controls. The raw data were deposited in the NCBI Gene Expression Omnibus database (https://www.ncbi.nlm.nih.gov/geo/query/acc.cgi?acc=GSE130795). A total of 19732 transcripts had detectable expression. We excluded most of the detectable transcripts according to the stringent screening criteria of a fold change ≥ 2.0 and a *p*-value < 0.05. Finally, 453 DE genes were identified. Among them, 276 were upregulated in CI-AKI, whereas the remaining 177 were downregulated (S5 Table). These included inflammation related genes *Gpnmb* (glycoprotein Nmb) [43] and *Cstb* (cystatin B) [44], cell death related gene *Gng7* (G protein subunit gamma 7) [45], as well as the renal protective gene *Gstm1* (glutathione S-transferase Mu 1) [46]. These specific genes and their functions merit further investigation. The DE genes were further visualized in a heat-map (Fig 4).

### Integrative analysis of putative miRNA-mRNA interactions

**Fig 5:**
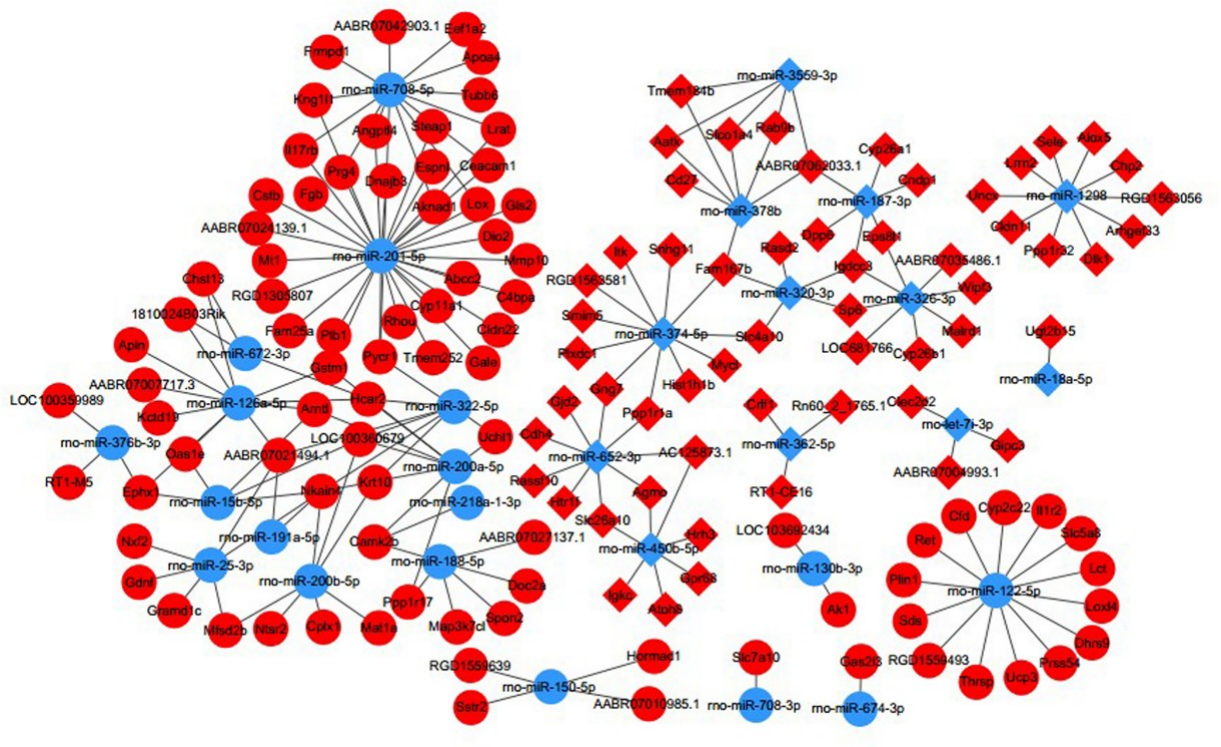
Integrated miRNA-mRNA analysis. The top 200 most negatively correlated putative miRNA-mRNA pairs (*P* < 0.05). Light blue circle indicates up-regulated miRNAs and red circle indicates down-regulated mRNAs. Light blue square indicates down-regulated miRNAs and red square indicates up-regulated mRNAs.

In the subsequent *in silico* analysis, a co-expression network of DE miRNAs and their target genes was performed to investigate miRNA-mediated post-transcriptional regulation in the development of CI-AKI. The potential relationships were predicted using TargetScan and miRanda. Consequently, 2037 miRNA-mRNA pairs with negative correlations, as well as 2746 pairs with positive correlations, were included in the network (S6 Table). Broadly speaking, upregulated miRNAs generally coincided with downregulated target mRNAs and vice versa. Therefore, those pairs where the miRNA and mRNA expression levels changed in the same direction were filtered out. We further focused on the 2037 negative miRNA-mRNA pairs, consisting of 35 mature miRNAs and 348 DE mRNAs (S1 Fig). The number of predicted targets for each miRNA ranged from 1 to 152. Only rno-miR-1247-5p was not significantly correlated with any target mRNA, and 105 mRNAs were not negatively targeted by any of the DE miRNAs. The top 200 most correlated pairs, composed of 25 dysregulated miRNAs and 112 dysregulated mRNAs, were visualized in Fig 5 (Pearson’s correlation coefficient > 0.961, *P* < 0.002).

Interestingly, a substantial overlap of correlated genes between different DE miRNAs was observed. A group of up-regulated genes (*Gpnmb*, *Lox* (lysyl oxidase), LOC100360679, *Hcar2* (hydroxycarboxylic acid receptor 2), *Apoa4* (apolipoprotein A4), *Mat1a* (methionine adenosyltransferase 1A), *Cldn22* (claudin 22), *Steap1* (STEAP family member 1), *Gstm1*, *Arntl* (aryl hydrocarbon receptor nuclear translocator like), *Ephx1* (epoxide hydrolase 1), and *Cstb*) were predicted to be targeted by each of the down-regulated miRNAs (rno-miR-378b, rno-miR-374-5p, rno-miR-652-3p, rno-miR-187-3p, rno-miR-320-3p, and rno-miR-3559-3p). Meanwhile, the up-regulated miRNAs (rno-miR-25-3p, rno-miR-30c-5p, rno-miR-126a-5p, rno-rno-miR-191a-5p, rno-miR-200a-5p, rno-miR-200b-5p, and rno-miR-322-5p) concurrently modulated a same group of down-regulated genes (*Tmem184b* (transmembrane protein 184B), *Cmah* (cytidine monophospho-N-acetylneuraminic acid hydroxylase), *Snhg11* (small nucleolar RNA host gene 11), *Cndp1* (carnosine dipeptidase 1), *Ppp1r1a* (protein phosphatase 1 regulatory inhibitor subunit 1A), *Prelid2* (PRELI domain containing 2), *Gng7*, Gjd2 (gap junction protein delta 2), *Smim5* (small integral membrane protein 5), *Itk* (IL2 inducible T cell kinase), *Rt1-db1* (RT1 class II, locus Db1), and *Rasd*2 (RASD family member 2)). The highly ranked miRNAs and genes in the networks were also presumed to be critical in the complex pathological process of CI-AKI.

### Validation using RT-qPCR

**Fig 6:**
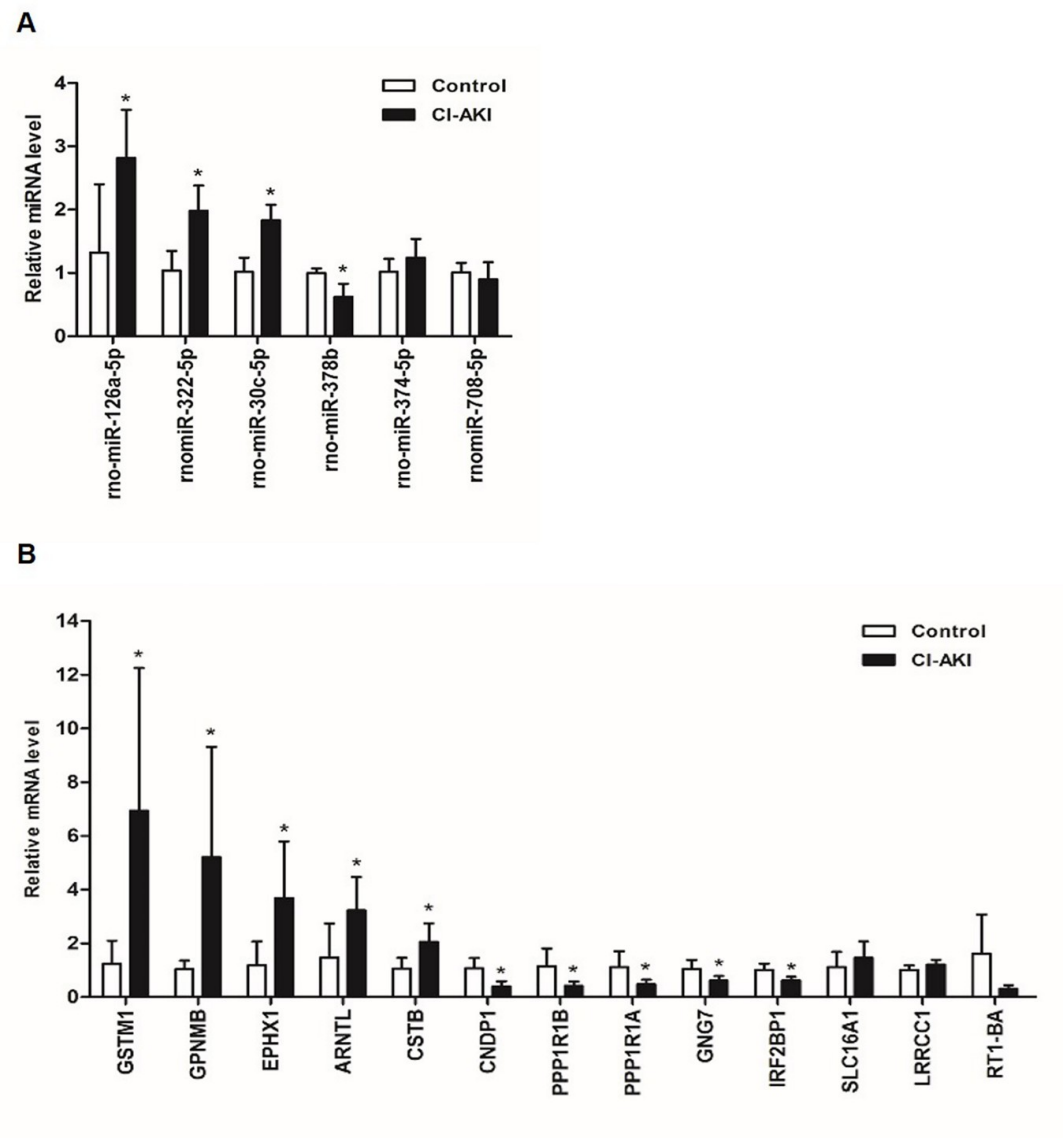
RT-qPCR validation. (A) RT-qPCR validation of six miRNAs selected from the miRNA sequencing data. N = 6 per group, *P < 0.05 vs. control group. (B) RT-qPCR validation of six mRNAs selected from the mRNA sequencing data. N = 6 per group, *P < 0.05 vs. control group.

**Fig 7:**
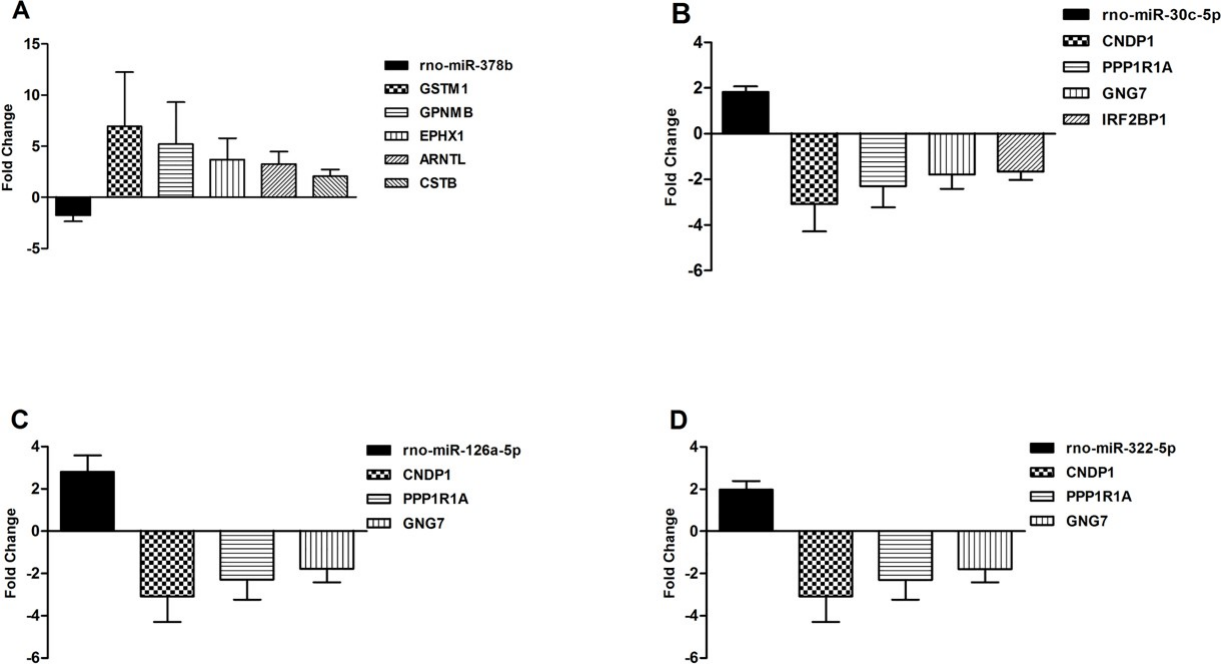
Putative negative miRNA-mRNA pairs validated by RT-qPCR (*P* < 0.05).

We selected miRNAs or mRNAs for further validation in the kidneys of six additional CI-AKI rats and six controls according to the following screening criteria: 1) The magnitude of their fold changes were significant between the groups; 2) the expression quantity should be adequate for successful detection via RT-qPCR; 3) they might play central roles by targeting multiple mRNAs or be targeted by multiple miRNAs; and 4) their previously identified functions were likely to be associated with CI-AKI. Consequently, a selected number of the DE mature miRNAs and 13 DE genes from the sequencing data were subsequently selected for RT-qPCR validation. Finally, four miRNAs (rno-miR-126a-5p, rno-miR-322-5p, rno-miR-30c-5p, and rno-miR-378b) and 10 genes (*Gstm1*, *Gpnmb*, *Ephx1*, *Arntl*, *Cstb*, *Cndp1*, *Ppp1r1b*, *Ppp1r1a*, *Gng7*, *and Irf2bp1* (interferon regulatory factor 2 binding protein 1)) that were demonstrated to be aberrantly expressed in CI-AKI showed RT-qPCR results that were in accordance with the sequencing results (Fig 6A and 6B, S7 Table). Negative correlations between the validated miRNAs and genes were analyzed (Fig 7, S8 Table).

## Discussion

CI-AKI is a serious complication in the clinical practice of coronary arteriography; however, its precise pathological mechanism remains obscure. The present study aimed to identify the transcriptional regulatory mechanisms in the rat kidney following CM injury.

To investigate this issue, we developed a reproducible rat model following intra-arterial CM injection to simulate the process of arteriography. However, the widely used low-osmolarity CM did not induce kidney injury in rats as easily as the high-osmolarity CM, despite pretreatment with indomethacin and L-NAME [8, 26]. Water deprivation can result in hypovolemia and the activation of the renin-angiotensin system, which predisposes the kidney to injury. Sun et al. reported that the addition of water dehydration for 3 days before the experiment was adequate [22]. However, we observed that a prolonged dehydration period of 3 days was associated with increased mortality of the rats during the experiment. A dehydration period of 48 hours proved to be optimal in our study, which might be partly attributed to the fact that an intra-arterial route of low-osmolarity CM is associated with a transient higher osmolality in the renal circulation than that achieved via the intravenous route [25]. In addition, we selected a timeline of 12 hours post CM injection to explore the miRNA-mRNA regulatory patterns, instead of 24 hours or 8 hours, as previously reported [22, 23]. First, a striking elevation of function-related SCr was detectable at 24 hours post surgery, indicating that the crest-time of kidney injury reaction had passed. Second, Sun et al. identified only 11 DE miRNAs in the kidney of CI-AKI rats at 8 hours post-procedure, using the same screening criteria as those used in the present study [22]. That study was designed to investigate early diagnosis, indicating that a timeline of 8 hours might be a little too early to explore the roles of miRNAs in the pathogenesis of CI-AKI. Additionally, a time-course study conducted by Gutierrez-Escolano et al. showed that expression changes of several key miRNAs peaked at 12 hours after CM injection [23]. Moreover, an adequate elevation of SCr over baseline had already been observed at 12 hours post-procedure in our present study. Thus, we believe that the timeline of 12 hours may have some advantages. Under these conditions, a reliable CI-AKI rat model with remarkable SCr elevation and pronounced histopathological changes was developed, which was comparable to human CI-AKI following coronary arteriography.

Based on this model, we conducted genome-wide miRNA and mRNA expression profiling analysis in the kidneys obtained from CI-AKI rats and controls using state-of-the-art RNA-seq technology. Specifically, we identified 36 miRNAs and 453 mRNAs that were differentially expressed in rat kidneys at 12 hours post-CM administration. In a further bioinformatic analysis, 2037 putative negatively correlated miRNA-mRNA pairs were revealed to be potentially implicated in the pathogenesis of this iatrogenic disease. To the best of our knowledge, this study provided the first temporal characterization of integrated miRNA-mRNA expression profiles in CI-AKI rats following intra-arterial CM injection.

The importance of dysregulated miRNAs in relation to kidney injury following CM exposure has been documented previously [22, 23]. Gutierrez-Escolano et al. initially reported that 51 miRNAs were differentially expressed in rat kidneys after intravenous administration of CM [23]. Correspondingly, the rat homolog of human miR-30c-5p was also identified to be upregulated in our current study. MiR-30c-5p, a member of miR-30 family, had been implicated in the pathogenesis of various kidney diseases. In a hypoxia-reoxygenation model, up-regulating miR-30c-5p was identified to protect tubular epithelial cell from apoptosis by targeting suppressor of cytokine signaling-3 [47]. Despite this beneficial role, Zhu et al. recently noted the significance of miR-30c-5p in facilitating the expression of reactive oxygen species [48], which are known to damage tubular and endothelial cells directly [49]. Moreover, miR-30c-5p was significantly elevated in the urine of rats and human patients following ischemia-reperfusion-induced kidney injury, reaching a peak level at 2 hours post-procedure [50]. Thus, we proposed that miR-30c-5p may play complicated roles in CI-AKI and be dysregulated at an early stage. In a recent study, Sun et al. highlighted 11 DE miRNAs in the CI-AKI kidney tissues [22], including the highly expressed rno-miR-188-5p, which was also identified in our current results. MiR-188-5p is likely to modulate inflammation and c-Jun N-terminal kinase and p38 mitogen-activated protein kinase pathways by targeting mitogen-activated protein Kinase kinase kinase 3 (MAP3K3) expression, indicating a potential role in CI-AKI [51–53]. The present study also revealed several other promising miRNAs, which had not been previously reported in CI-AKI. Among them, miR-322-5p was recently reported to putatively target insulin-like growth factor 1, a factor known to protect cells from stress-induced apoptosis, leading to a pro-apoptosis effect [54, 55]. MiR-378b was observed to be down-regulated in carbon-tetrachloride treated mice and was suspected to promote liver fibrosis [56]. MiR-126a-5p was reported to stimulate epithelial-mesenchymal transdifferentiation [57], a hallmark of kidney fibrosis and the development of human chronic kidney disease [58]. The underexpressed rno-miR-378b and the overexpressed rno-miR-126a-5p may be associated with kidney fibrosis, leading to persistent renal damage in patients with CI-AKI [6].

As indicated in the KEGG enrichment analysis of these DE miRNAs, CM significantly increased several pathways in the rat kidney, including the AMPK, mammalian target of rapamycin (mTOR), Wnt, and TGF-β signaling pathways. AMPK and mTOR are two important energy-sensing pathways in the kidney. Both pathways are closely related to mitochondrial homeostasis and autophagy, in response to hypoxia, energy depletion, and oxidative stress [59]. Recently, CM was observed to contribute to the generation of reactive oxygen species and mitophagy in renal tubular epithelial cells in vitro [60]. The miRNAs implicated in these pathways might play important regulatory roles in oxidative stress-mediated mitochondrial dysfunction and mitophagy, a significant pathological process underlying CI-AKI [8]. The Wnt signaling pathway is recognized to promote repair and regeneration after acute kidney injury and facilitates the progression of chronic kidney disease [40]. This pathway may also modulate apoptosis of tubular epithelial cells during acute kidney injury induced by ischemia-reperfusion or cisplatin [61]. TGF-β signaling might induce profibrotic and protective effects as a response to kidney injury [41]. Taken together, the KEGG pathway analysis suggested that the DE miRNAs are potentially involved in several key pathways of CI-AKI development. Particularly, rno-miR-322-5p and rno-miR-378b were implicated in all of the important pathways mentioned above, indicating that they may have central roles in the regulation of CI-AKI. These miRNAs may serve as new targets for therapeutic intervention.

Additionally, we also characterized multiple DE mRNAs that might be essential for the pathogenesis of CI-AKI. For instance, *Cstb* is a ubiquitous cysteine cathepsin that has been extensively studied in non-alcoholic steatohepatitis. Tang et al. observed recently that *Cstb* might facilitate inflammation and apoptosis by promoting the expression and activity of caspase-1, as well as the secretion of IL-1β and IL-18 [44]. *Ephx1* levels were observed to be significantly elevated in the rat kidney following cisplatin treatment, although the function of this protein was not further elucidated [62]. Correspondingly, a large number of DE genes are connected to protective reactions against kidney injury. Among the overexpressed genes was *Gpnmb* (fold change = 3.61), which was observed to be highly expressed in macrophages following renal ischemic-reperfusion injury [43]. Upregulated *Gpnmb* was suspected to protect the kidney from injury by modulating the polarization of macrophages. *Gng7* could induce autophagy and cell death by inhibiting the mTOR pathway [45], and the reduced of this gene in our results might indicate a cytoprotective effect. These findings suggested that even in the relatively early stage of CI-AKI, the adaptive repair and recovery processes are already under way.

The bioinformatics analysis produced a library of putative miRNA-mRNA regulatory interactions. For example, rno-miR-378b was down-regulated in CI-AKI rats. Meanwhile, the over-expression of multiple transcripts (*Gstm1*, *Gpnmb*, *Ephx1*, *Arntl*, and *Cstb*) was negatively correlated with rno-miR-378b. The down-regulated genes (*Cndp1*, *Ppp1r1a*, *Gng7*, and *Irf2bp1*), were putatively targeted by the up-regulated rno-miR-30c-5p. Notably, we observed that a group of miRNAs might commonly regulate a group of genes. In other words, each of the genes in the same set was negatively correlated with all of the members of a certain group of miRNAs, and conversely, each miRNA might putatively target a group of genes. Briefly, our results suggested the complexity of the genetic networks controlling pathogenesis of CI-AKI, which to date has remained largely unexplored.

Despite the significant findings reported in this study, several limitations must also be recognized. First, we did not investigate the dynamic and tissue-specific expression profiles of the dysregulated miRNAs and genes. Thus, it remains unclear which miRNAs and genes are more crucial or specific in the kidney, or whether they exhibit the greatest effects. Second, the novel DE miRNAs identified using high-throughput sequencing were not included in our analysis. The large amounts of data generated require further excavation. Third, the high false-positive rate of the algorithms defining the relevant miRNA-mRNA interactions should also be considered. Thus, more studies are needed to verify the putative miRNA-mRNA pairs in CI-AKI, as well as the interaction mechanisms and downstream signaling pathways.

### Conclusions

In summary, our study identified the expression profiles of both miRNAs and mRNAs in a CI-AKI rat model following intra-arterial CM injection. We revealed a number of DE miRNAs and mRNAs that might be related to CI-AKI, and further illustrated a putative miRNA-mRNA regulatory network. Our results highlighted that the dysregulated miRNAs, as well as their potential targets, might be involved in various aspects of the pathology of CI-AKI, including inflammation, energy metabolism, oxidative stress reaction, cell proliferation and death, and interstitial fibrosis. Based on the integrated analysis, our findings provide the basis for further investigations of the pathogenesis of CI-AKI.

## Supporting information

S1 Table Changes of serum creatinine levels of the experimental rats. (DOCX)

S2 Table Differentially expressed miRNAs between CI-AKI rats and controls. (XLS)

S3 Table GO enrichment analysis of differentially expressed miRNAs. (XLS)

S4 Table KEGG enrichment analysis of differentially expressed miRNAs. (XLS)

S5 Table Differentially expressed mRNAs between CI-AKI rats and controls. (XLS)

S6 Table miRNA-mRNA integrated analysis. (XLS)

S7 Table qRT-PCR validation of selected DE miRNAs and mRNAs. (DOCX)

S8 Table Putative negative miRNA-mRNA pairs validated by qRT-PCR. (DOCX)

S1 Fig Negatively correlated putative miRNA-mRNA pairs. (JPG)
