# Supplementary material for: Co-expression analysis reveals dysregulated miRNAs and miRNA-mRNA interactions in the development of contrast-induced acute kidney injury
Source: PLoS One. 2019 Jul 15;14(7):e0218574. doi: 10.1371/journal.pone.0218574 (PMC6629072; doi:10.1371/journal.pone.0218574)
Supplement: S1 Table — (DOCX) [file pone.0218574.s001.docx]

**S1 Table.** **Characteristics of the experimental rats**

| Serial No. | Body weight (g) | Baseline sCr (μmol/L) | Increasing amplitude of sCr (μmol/L) | Percent increment of sCr (%) | Median Paller score from 10 highmagnification (×200) fields |
| --- | --- | --- | --- | --- | --- |
| Control 1 | 380 | 42.0 | 29.0 | -28.6 | 4 |
| Control 2 | 390 | 56.0 | 40.0 | -28.6 | 5 |
| Control 3 | 370 | 44.0 | 36.0 | -18.2 | 2 |
| Control 4 | 310 | 41.0 | 35.0 | -14.6 | 2 |
| Control 5 | 384 | 55.0 | 64.4 | 17.1 | 3 |
| Control 6 | 376 | 59.0 | 58.7 | -0.5 | 3 |
| Control 7 | 387 | 68.0 | 51.0 | -25.0 | 5 |
| Control 8 | 400 | 55.0 | 61.8 | 12.4 | 3 |
| Control 9 | 340 | 60.0 | 55.0 | -8.3 | 5 |
| Case 1 | 350 | 49.0 | 94.0 | 91.8 | 10 |
| Case 2 | 360 | 40.4 | 74.0 | 81.2 | 8 |
| Case 3 | 300 | 41.0 | 72.0 | 75.6 | 8 |
| Case 4 | 340 | 58.0 | 77.7 | 33.3 | 7 |
| Case 5 | 300 | 24.0 | 44.0 | 83.3 | 8 |
| Case 6 | 395 | 58.0 | 89.1 | 53.6 | 8 |
| Case 7 | 300 | 57.0 | 80.0 | 40.4 | 8 |
| Case 8 | 385 | 36.2 | 49.0 | 35.4 | 7 |
| Case 9 | 350 | 31.9 | 46.0 | 44.2 | 9 |
