# Supplementary material for: Co-expression analysis reveals dysregulated miRNAs and miRNA-mRNA interactions in the development of contrast-induced acute kidney injury
Source: PLoS One. 2019 Jul 15;14(7):e0218574. doi: 10.1371/journal.pone.0218574 (PMC6629072; doi:10.1371/journal.pone.0218574)
Supplement: S7 Table — (DOCX) [file pone.0218574.s007.docx]

**S8 Table. Relative expression of selected differentially expressed miRNAs and mRNAs through qRT-PCR**

| miRNA/miRNA | Relative miRNA/mRNA level (Mean ± SD) | | Regulation | *P* value |
| --- | --- | --- | --- | --- |
|  | Control | CI-AKI |  |  |
| rno-miR-126a-5p | 1.32 ± 1.08 | 2.82 ± 0.76 | Up | 0.019 |
| rno-miR-322-5p | 1.04 ± 0.31 | 1.98 ± 0.40 | Up | 0.001 |
| rno-miR-30c-5p | 1.02 ± 0.22 | 1.83 ± 0.25 | Up | < 0.001 |
| rno-miR-378b | 1.00 ± 0.07 | 0.62 ± 0.21 | Down | 0.002 |
| rno-miR-374-5p | 1.02 ± 0.20 | 1.24 ± 0.30 | Up | 0.165 |
| rno-miR-708-5p | 1.01 ± 0.15 | 0.90 ± 0.27 | Down | 0.408 |
| GSTM1 | 1.24 ± 0.86 | 6.94 ± 5.32 | Up | 0.027 |
| GPNMB | 1.04 ± 0.31 | 5.21 ± 4.10 | Up | 0.032 |
| EPHX1 | 1.18 ± 0.89 | 3.68 ± 2.11 | Up | 0.023 |
| ARNTL | 1.47 ± 1.27 | 3.23 ± 1.24 | Up | 0.036 |
| CSTB | 1.06 ± 0.40 | 2.05 ± 0.70 | Up | 0.013 |
| CNDP1 | 1.07 ± 0.38 | 0.39 ± 0.20 | Down | 0.003 |
| PPP1R1B | 1.15 ± 0.66 | 0.42 ± 0.15 | Down | 0.024 |
| PPP1R1A | 1.12 ± 0.58 | 0.48 ± 0.16 | Down | 0.028 |
| GNG7 | 1.04 ± 0.34 | 0.61 ± 0.17 | Down | 0.017 |
| IRF2BP1 | 1.02 ± 0.22 | 0.62 ± 0.13 | Down | 0.003 |
| SLC16A1 | 1.12 ± 0.56 | 1.46 ± 0.61 | Up | 0.334 |
| LRRCC1 | 1.01 ± 0.16 | 1.21 ± 0.18 | Up | 0.063 |
| RT1-BA | 1.62 ± 1.45 | 0.31 ± 0.13 | Down | 0.052 |
