# Supplementary material for: Co-expression analysis reveals dysregulated miRNAs and miRNA-mRNA interactions in the development of contrast-induced acute kidney injury
Source: PLoS One. 2019 Jul 15;14(7):e0218574. doi: 10.1371/journal.pone.0218574 (PMC6629072; doi:10.1371/journal.pone.0218574)
Supplement: S8 Table — (DOCX) [file pone.0218574.s008.docx]

**S9 Table.** **Putative negative miRNA-mRNA pairs validated by qRT-PCR**

| miRNAs | Fold change  (mean ± SD) | mRNAs | Fold change  (mean ± SD) | Correlation | *P* value |
| --- | --- | --- | --- | --- | --- |
| rno-miR-378b | -1.76 ± 0.57 | GSTM1 | 6.94 ± 5.32 | -0.99 | <0.001 |
| rno-miR-378b | -1.76 ± 0.57 | GPNMB | 5.21 ± 4.10 | -0.97 | 0.002 |
| rno-miR-378b | -1.76 ± 0.57 | EPHX1 | 3.68 ± 2.11 | -0.96 | 0.003 |
| rno-miR-378b | -1.76 ± 0.57 | ARNTL | 3.23 ± 1.24 | -0.96 | 0.003 |
| rno-miR-378b | -1.76 ± 0.57 | CSTB | 2.05 ± 0.70 | -0.96 | 0.002 |
| rno-miR-30c-5p | 1.83 ± 0.25 | CNDP1 | -3.08 ± 1.21 | -0.92 | 0.008 |
| rno-miR-30c-5p | 1.83 ± 0.25 | PPP1R1A | -2.30 ± 0.93 | -0.92 | 0.010 |
| rno-miR-30c-5p | 1.83 ± 0.25 | GNG7 | -1.79 ± 0.62 | -0.96 | 0.002 |
| rno-miR-30c-5p | 1.83 ± 0.25 | IRF2BP1 | -1.67 ± 0.35 | -0.94 | 0.005 |
| rno-miR-126a-5p | 2.82 ± 0.76 | CNDP1 | -3.08 ± 1.21 | -0.95 | 0.004 |
| rno-miR-126a-5p | 2.82 ± 0.76 | PPP1R1A | -2.30 ± 0.93 | -0.94 | 0.006 |
| rno-miR-126a-5p | 2.82 ± 0.76 | GNG7 | -1.79 ± 0.62 | -0.95 | 0.003 |
| rno-miR-322-5p | 1.98 ± 0.40 | CNDP1 | -3.08 ± 1.21 | -0.93 | 0.006 |
| rno-miR-322-5p | 1.98 ± 0.40 | PPP1R1A | -2.30 ± 0.93 | -0.98 | <0.001 |
| rno-miR-322-5p | 1.98 ± 0.40 | GNG7 | -1.79 ± 0.62 | -0.97 | 0.001 |
