# Supplementary figures and images for: Co-expression analysis reveals dysregulated miRNAs and miRNA-mRNA interactions in the development of contrast-induced acute kidney injury
Source: PLoS One. 2019 Jul 15;14(7):e0218574. doi: 10.1371/journal.pone.0218574 (PMC6629072; doi:10.1371/journal.pone.0218574)

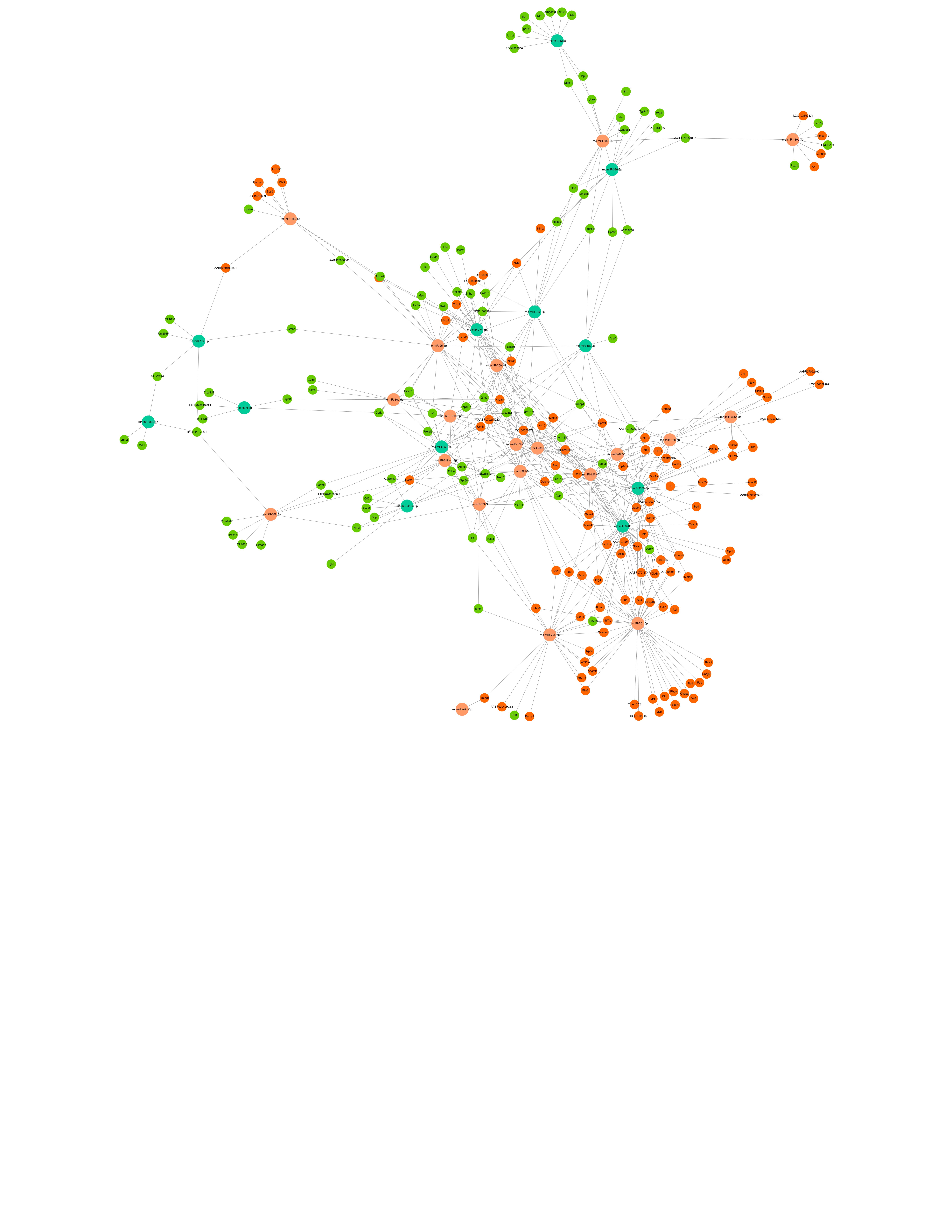

Supplement: S1 Fig — (JPG) [file pone.0218574.s009.jpg]
